# Electing amateur politicians reduces cross-party collaboration

**DOI:** 10.1073/pnas.2519787122

**Published:** 2025-10-09

**Authors:** Rachel Porter, Jeffrey J. Harden, Mackenzie R. Dobson

**Affiliations:** ^a^Department of Political Science, University of Notre Dame, Notre Dame, IN 46556; ^b^Department of Politics, University of Virginia, Charlottesville, VA 22904

**Keywords:** bipartisanship, Congress, political experience, polarization

## Abstract

Public trust in democratic institutions has dropped to historic lows, prompting electorates in major democracies to turn to “amateur” politicians with the expectation that these political outsiders will cut through stalemates to deliver policy results. Amateurs are often seen as pragmatic “doers,” but also uncompromising, a combination at odds with governing systems where legislative progress depends on cross-party coalitions. Using the US Congress as a critical case, we evaluate these competing expectations by linking over four decades of election data with 2.2 million bill (co)sponsorship records. We find that electing amateurs intensifies partisan divisions: Districts that send amateurs to Congress yield representatives who attract fewer opposing-party collaborators to their bills and less often support other-party legislation. Our results suggest that amateurs are unlikely to deliver on their promise for pragmatic governance, as they resist or undervalue the compromises essential to lawmaking.

In 2016, political novice Donald Trump won the US presidency, and in the years that followed, waves of other amateurs—individuals with no prior experience holding elective office—entered the US Congress. This rise reflects a broader trend: Major democracies are electing amateurs to legislatures at unprecedented rates (1–3). The surge in amateur success stems from widespread disillusionment with traditional governance. Cross-national surveys find that majorities perceive their governments as incapable of addressing policy crises (4), which has eroded trust in representative institutions, such as legislatures and political parties (5).

Amateurs are cast as agents of political renewal, capable of breaking through “the establishment” and “insider politics” to address policy challenges. In this way, they contrast with career politicians, who are perceived as corrupt, out of touch, and ineffective at delivering policy outcomes (4). Yet scholarship shows that because amateurs emerge through activist rather than party networks, they tend to exhibit more ideological behavior than experienced candidates, who advance through the conventional political career ladder, starting in lower elective positions (6, 7). In governing systems with numerous veto players, the rigidity of ideological commitment often conflicts with legislative effectiveness, as policy progress depends on cross-party bargaining and coalition-building (8, 9). The worldwide rise of amateurs underscores the importance of evaluating their collaborative capacity; yet, systematic evidence adjudicating whether these political outsiders contribute to pragmatic lawmaking and effective governance remains scarce.

We provide an empirical analysis examining the consequences of electing amateurs for cross-party cooperation in the US House of Representatives. Our theoretical perspective emphasizes how amateurs’ lack of elective experience shapes their legislative behavior. Specifically, we argue that prior service in elected roles equips legislators with the experience of navigating cross-party relationships, thereby fostering a greater propensity for bipartisan collaboration. Amateurs, by contrast, enter Congress without such experience. Lacking exposure to the practical value of compromise, they are more likely to view cross-party cooperation as an undesirable concession rather than a necessary condition for lawmaking.

Our analyses employ comprehensive data on candidates’ office-holding experience (3) and electoral outcomes in US House races from 1980 to 2022 (10), linked to an original dataset of over 2.2 million (co)sponsorship records. We pair these data with two research designs: 1) a regression discontinuity design that identifies the causal effect of narrowly electing amateurs over incumbents on subsequent bipartisan legislative collaboration, and 2) a panel data estimation of the contrast between district representation by a member of Congress (MC) who entered the chamber without prior elected experience versus a congressperson who entered with experience holding elective office. Our results show that electing amateur politicians does not improve bipartisan cooperation; rather, it significantly reduces collaboration between parties. Compared to districts represented by incumbents and/or legislators with prior elective experience, those served by amateurs see significantly less bipartisan coalition building. Specifically, amateurs both attract fewer cross-party collaborators to their legislation and offer less support to opposing-party bills.

## Materials and Methods

### Data.

We integrate multiple data sources to examine the relationship between candidate experience and legislative behavior. Electoral data encompasses general election returns for US House districts from 1980 to 2022 (10), totaling 10,441 races and 19,619 major-party candidates. These election data are supplemented with candidate experience information (3), documenting prior service in elected positions within state legislatures (e.g., state senate), municipal offices (e.g., mayor), and statewide positions (e.g., secretary of state). We complete the dataset with records we collected on the legislative activity of House members across the 97th through 118th Congresses. Specifically, we construct our outcome measures from data spanning 145,100 instances of bill sponsorship and 2,220,735 cosponsorships. We limit our measures to only instances of original cosponsorship—defined as the formal endorsement of a legislative colleague’s bill at the time of its introduction. Our measures of bipartisanship are defined as follows:

- Bipartisan Original Cosponsorships Attracted: the proportion of original cosponsors on a legislator’s bill who are from the opposing party, on average, across all bills the legislator sponsored in a given two-year session.
- Bipartisan Original Cosponsorships Offered: the proportion of bills for which a legislator was an original cosponsor that members of the opposing party introduced in a given session.^*^

### Regression Discontinuity Design.

The regression discontinuity design (RDD) leverages candidates’ two-party vote shares—the percentage of votes received by the nonincumbent candidate relative to the total votes cast for the two major parties—in general elections as a “forcing variable.” Treatment assignment occurs as-if randomly at the 50% vote threshold, distinguishing barely victorious amateurs from narrowly defeated incumbents (11). As such, this design only encompasses elections in which an amateur ran against an incumbent member (N = 5,875). We assume districts electing these candidate types are exchangeable; in *SI Appendix*, we discuss empirical diagnostics testing this assumption. The key quantity is the local average treatment effect (LATE) of a district electing an amateur instead of an incumbent in US House general elections (12). We estimate this effect on bipartisanship in the immediate two-year congressional term and two subsequent terms (i.e., six postelection years).^†^

### Panel Data Design.

In our panel data design, congressional districts serve as the units, and two-year terms are the time periods. An indicator variable denotes terms in which a district is represented by a member who entered Congress as either an amateur (no prior elected experience) or an experienced politician. We rely on two panel data methods that estimate the average treatment effect on the treated (ATT), each with different underlying assumptions: PanelMatch (13) and FEct (14). See the *SI Appendix* for summaries of these approaches. With both methods, we employ covariates to mitigate confounding, including candidate characteristics (e.g., ideology and demographics), district factors (e.g., redistricting and constituency characteristics), and institutional context (e.g., partisan majority size). Although these panel design approaches do not benefit from the identification advantages inherent to the RDD discussed above, their specification allows for the inclusion of a more extensive sample of districts (N = 9,432) and the explicit comparison between new members who were amateurs prior to entering Congress and those who were experienced office-holders.

## Results

**Fig. 1.**
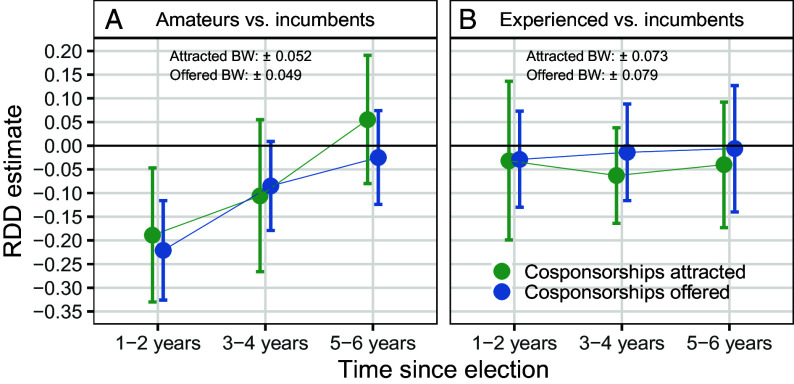
Effects of electing (*A*) an amateur and (*B*) an experienced challenger instead of an incumbent on bipartisan original cosponsorship in Congress over time.

Panel (*A*) of Fig. 1 reports treatment effect estimates and 95% CIs from the RDD on the bipartisanship outcomes in three future congressional terms after the election of an amateur: the immediate term (1 to 2 y after election), second term (3 to 4 y), and third term (5 to 6 y). We employ a local linear regression specification, a mean squared error (MSE) optimized bandwidth (BW) selector, and a triangular kernel. The graphs also present the chosen bandwidths as deviations from a tied election (e.g., ±0.052). See *SI Appendix* for alternative specifications.

The results indicate that when a district narrowly elects an amateur over an incumbent, that district yields a representative who attracts cross-party collaborators to their legislation at a lower rate (19 percentage point decrease) and offers bipartisan support to other-party colleagues’ legislation less frequently (22 percentage point decrease). These effects are sizable, equivalent to 81% and 127% of a SD in the respective outcomes. On average, legislators in our sample attract five other-party original cosponsors to their bills per term and offer bipartisan original cosponsorship on 17 bills. Estimates in Fig. 1, panel (*A*) suggest that electing an amateur eliminates approximately one of the five opposing-party cosponsors per bill and results in about four fewer bipartisan cosponsorships offered compared to the counterfactual of the district retaining the incumbent.

In the second term after a district elects an amateur, the effects are no longer statistically distinguishable from zero but remain negative and substantively meaningful, reflecting an 11-point reduction in attracted cosponsorships and nine-point reduction in offered support. These effect sizes represent 45% and 49% of a SD in their respective outcomes. Their magnitudes are stronger than the deterioration in bipartisanship observed among MCs who voted against certifying the 2020 presidential election on January 6, 2021 (15). By the third term, however, the district’s representative is indistinguishable—both statistically and substantively—from a retained incumbent. This pattern suggests a learning process, with amateurs initially underperforming but gaining collaborative skills over time.

One alternative explanation for the results in panel (*A*) of Fig. 1 is a freshman effect: The observed decline in bipartisan representation a district encounters may reflect its member’s lack of congressional experience, rather than amateur status. We examine this possibility in panel (*B*), which reports the same RDD but contrasts districts that narrowly elect an experienced challenger with those that retain an incumbent (N = 1,345). We expect that experienced freshmen members, having served within state and/or municipal governments, are equipped with a stronger appreciation for the value of cross-party cooperation and compromise. If panel (*A*) reflects a reduction in bipartisan representation among districts electing any new member, we should expect to see similar results for this experienced challenger versus incumbent contrast in panel (*B*).

Fig. 1, panel (*B*) suggests a different conclusion regarding the RDD effects for districts electing experienced challengers relative to retaining incumbents. The point estimates are slightly negative (median of −0.03), but remain relatively flat over time, unlike the upward trend observed in panel (*A*). Across all time periods, the estimates are not statistically distinguishable from zero. Districts electing new members with prior elective experience receive similar degrees of bipartisan representation relative to those retaining incumbents. Overall, Fig. 1 implies that the penalty to bipartisan collaboration is unique to districts electing amateur politicians who lack valuable prior experience, a contrast we explore in greater detail next.

### A Broader and Direct Comparison.

Although the RDD yields considerable causal leverage, it also suffers from two key shortcomings. First, it estimates the district-level effects of barely electing an amateur or an experienced challenger instead of an incumbent. These scenarios cover a limited set of cases. Second, due to research design constraints, we cannot directly compare amateurs to experienced new members using the RDD.^‡^ Accordingly, we next pair the complete set of district-terms in our sample with PanelMatch and FEct to estimate the effect of representation by an amateur politician relative to a member with an office-holding background on our bipartisanship outcomes, controlling for member seniority and other relevant factors.

**Fig. 2.**
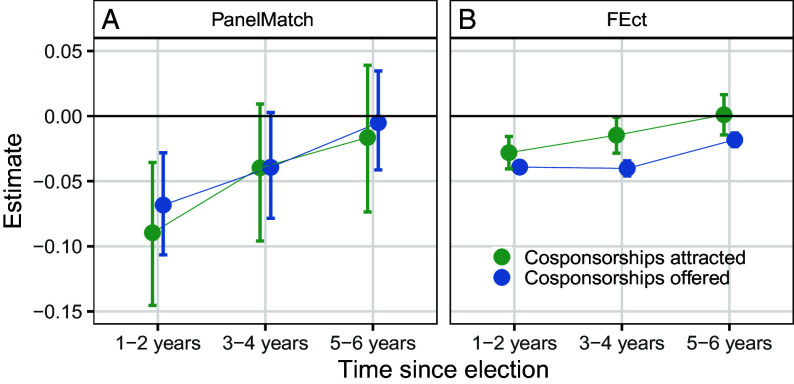
Effect of an amateur politician serving in a district instead of an experienced new member on bipartisan original cosponsorship in Congress over time, for (*A*) PanelMatch and (*B*) FEct estimators.

Fig. 2 shows that bipartisan original cosponsorship among amateur freshmen is notably lower compared to experienced freshmen in the first postelection term—by 7 to 9 (3 to 4) percentage points with PanelMatch (FEct). These differences shrink toward zero in the subsequent two terms, although FEct still detects statistically distinguishable penalties to offered cosponsorships of approximately 4 percentage points in the second term and two points in the third. In short, employing a broader set of cases and specifying a design that directly compares districts represented by amateurs versus experienced new members, we again find that electing an amateur politician is associated with declines in cross-party cosponsorship activity.

## Conclusions

Our investigation provides strong evidence that electing amateur politicians to the US Congress significantly undermines bipartisan cooperation. The negative effects identified across multiple research designs are substantively large, exceeding those associated with other established predictors of bipartisan cosponsorship.^§^ They are comparable to the impact of one of the most serious breaches of democratic norms in recent US history—some Republican legislators’ refusal to certify the 2020 presidential election results (15)—highlighting the major threat that amateurs pose to effective governance. The continual influx of new amateurs means that their negative effects on bipartisanship recur across election cycles (3). These challenges grow sharper as more amateurs today achieve success, including figures like Alexandria Ocasio-Cortez (D-NY) and Marjorie Taylor Greene (R-GA), who have built careers on rejecting compromise.

Our findings specifically reflect the polarized environment of the US Congress. Further research is necessary to examine how collaboration dynamics unfold in other countries experiencing spikes in amateur representation, as their different institutional designs may yield divergent outcomes (8). Prominent examples include Italy’s Five Star Movement, France’s En Marche! (founded by Emmanuel Macron), Ukraine’s Servant of the People Party (led by Volodymyr Zelenskyy), and the recent election of Canadian Prime Minister Mark Carney. Collectively, these examples demonstrate the importance of assessing the implications of amateurism across diverse institutional contexts.

Our findings highlight the tension between the popular expectation of amateurs as agents of renewal and their practical tendency to intensify partisan division and resist compromise. These dynamics highlight the need for closer examination of how outsider politicians reshape the relationship between citizens and representative institutions. Our study provides an empirical foundation for these debates and their implications for democratic governance.

## Supplementary Material

Appendix 01 (PDF)

## Data Availability

Replication data and code files are available in the Harvard Dataverse (16).

## Footnotes

^*^In *SI Appendix*, we discuss analyses with total cosponsorships as the dependent variable. In nearly all cases, effects are near zero and/or not statistically distinguishable from zero, indicating that districts electing amateurs to Congress do not experience a decline in cosponsorship more broadly.

^†^In cases in which a legislator served in the immediate term, but not the second or third, we code the outcomes based on the legislator who did serve in the later term(s) for that district. This approach avoids posttreatment bias from nonrandom attrition rates between amateurs and incumbents.

^‡^Validity diagnostics (e.g., sorting at the threshold) fail on the small sample of open-seat races featuring amateurs/experienced candidates, making the panel design the optimal approach.

^§^In *SI Appendix*, we conduct a meta-analysis of studies predicting bipartisanship from politician traits.
