# Supplementary material for: Electing amateur politicians reduces cross-party collaboration
Source: Proc Natl Acad Sci U S A. 2025 Oct 9;122(41):e2519787122. doi: 10.1073/pnas.2519787122 (PMC12541438; doi:10.1073/pnas.2519787122)
Supplement: Supplementary file 1 — Appendix 01 (PDF) [file pnas.2519787122.sapp.pdf]

Supporting Information for

Electing amateur politicians reduces cross-party collaboration

Rachel Porter, Jeffrey J. Harden, Mackenzie R. Dobson

Rachel Porter.
E-mail: rachel.porter@nd.edu

This PDF file includes:

Supporting text
SI References

Supporting Information Text

Extended Materials and Methods

To examine the legislative implications of electing amateur politicians to the U.S. House of Representatives, we leverage and expand upon multiple data sources detailing candidate backgrounds, election outcomes, and legislative behavior. We then employ two complementary research designs to estimate the effect of a district electing a political amateur (i.e., a candidate with no prior experience holding elective office) on subsequent bipartisan original cosponsorship activity.

Data. Election results: General election returns for all U.S. House of Representatives districts (1980-2022) come from the MIT Election Data and Science Lab (https://dataverse.harvard.edu/citation?persistentId=doi:10.7910/DVN/IG0UN2). This dataset comprises 10,441 individual races involving 19,619 major-party candidates. We calculate vote shares for the top-performing Democrat and Republican in each general election. In states permitting fusion voting, such as New York and Connecticut, we aggregate votes for major-party candidates listed under multiple party lines (e.g., Democratic Party and Working Families Party). Additionally, we classify candidates affiliated with Minnesota's Democratic–Farmer–Labor Party as Democrats.

Candidate elective experience: Data on U.S. House candidates' elective experience is sourced from established research (1, 2). We expand upon these existing data by coding the elective backgrounds of candidates excluded from extant data, such as candidates who participated in off-cycle special elections and the 2022 general election (N = 1,152). Our coding process adheres closely to previously established protocols (1, 2), classifying candidates as *experienced* if they currently hold or have previously held a publicly elected office. Specifically, we categorize U.S. House candidates who have served as state representatives, state senators, governors, or members of Congress as experienced, excluding rare cases involving appointments to these positions. Candidates who have held other state or local elected offices—such as city council members, aldermen, sheriffs, or treasurers—are similarly classified as experienced, provided their positions were obtained via election. Notably, certain political offices differ across states in terms of elective or appointive status (e.g., the Secretary of State is elected in Georgia but appointed in Texas). To accurately distinguish elected from appointed positions, we verified candidates' elective histories through careful examination of media coverage, official ballot records, and state documents.

We employed a comprehensive, multi-source approach to identify candidates' prior elective experience. For candidates who ran in elections held in the 1980s and 1990s, we primarily relied on advanced archival searches of local newspapers accessed through Newspapers.com. For candidates who ran in more modern elections, we additionally searched digital news articles indexed by Google News. These searches were geographically and temporally constrained to relevant election cycles and further refined with keywords such as "Congress" and "election." Through these procedures, we successfully documented the prior elective experience of all candidates lacking information in existing datasets.

Congressional district characteristics: Data on district characteristics, such as average presidential vote-share, primary election rules, and seat vacancy status, are sourced mainly from extant work (2). We updated these data, which cover 1980 to 2020, to include district characteristics for 2022, incorporating estimates of 2020 presidential vote shares adjusted to new district boundaries after redistricting generated by The Downballot (https://www.the-downballot.com/p/the-downballots-calculations-of-presidential) and information on state primary election rules from the National Council of State Legislatures (NCSL; https://www.ncsl.org/elections-and-campaigns/2022-state-primary-election-dates-and-filing-deadlines).

Candidate fundraising and ideology: Measures of candidate ideology and campaign fundraising are obtained from the Database on Ideology, Money in Politics, and Elections (DIME; https://data.stanford.edu/dime), which compiles and standardizes all itemized political contributions to U.S. House candidates from 1980 to 2022. Under the assumption that donors exhibit spatial proximity in their contribution behavior, DIME estimates candidates' one-dimensional ideological ideal points based on the structure of the donor–recipient network (3).

Bill sponsorship and cosponsorship: Data on bill sponsorship and cosponsorship in the U.S. House for the 97th through 118th Congresses come from Congress.gov—the official government website for federal legislative information. We queried the Congress.gov API for metadata on all U.S. House bills introduced in each Congress. We did not collect data on simple, concurrent, or joint resolutions. Data retrieval via this API took approximately 85 computing hours due to rate limits restricting hourly queries and data pagination that resulted in multiple queries on bills with extended lists of cosponsors. The resulting dataset comprises two relational structures: one capturing unique bill-sponsor pairings and another capturing unique bill-cosponsor pairings. Metadata provided by Congress.gov includes a dichotomous indicator for whether or not a given cosponsorship was an original cosponsorship.

Additional Metadata: Information on congressional district characteristics—including total population, median income, and racial composition—are sourced from the U.S. Census American Community Survey (ACS; https://www.census.gov/programs-surveys/acs.html). Data on legislators' leadership roles, committee chair assignments, gender, and race are drawn from the Center for Effective Lawmaking database (CEL; https://thelawmakers.org/data-download).

Merging data. To create a harmonized dataset at the congressional district level, we first employed deterministic merging methods based on exact string matches of election year, state, district number, and partisan affiliation. However, integrating candidate-level election results with bill sponsorship and cosponsorship records required more advanced linkage techniques due to the absence of standardized candidate identifiers. Geographic identifiers alone (state and congressional district) do not uniquely determine candidate identities. Inconsistencies in naming conventions, such as nicknames and suffixes (e.g., George Joseph Kelly Jr. of Pennsylvania's 16th district, commonly known as Mike Kelly), prevent exact string matching. To address this issue, we implemented a probabilistic record linkage framework using pretrained name embeddings to generate pairwise

similarity scores between candidate names (4). To improve efficiency and accuracy, we applied blocking by state, district, and election year. This strategy successfully matched 93% of candidate records in our dataset, with the remaining unmatched cases resolved through manual adjudication.

Outcome measures. We constructed outcome measures using sponsorship and cosponsorship data for all bills introduced from the 97th through the 118th Congresses. The resulting dataset encompasses 145,100 bill sponsorships and 2,220,735 cosponsorships. We limited our analysis to bills that received at least one original cosponsorship—defined as a cosponsor who formally supports a bill at the time of its introduction—and excluded any withdrawn cosponsorships from our measures. Typically, each bill can have only one primary sponsor, though any number of cosponsors may join. A notable exception occurred on January 4, 1995, during the 104th Congress when twelve bills were introduced with multiple primary sponsors pursuant to H.Res. 6. For these cases, we created distinct bill-sponsor pairings for each primary sponsor, treating all additional sponsors as original cosponsors in our outcome measures.

Our measure for *Bipartisan Original Cosponsorships Attracted* is the proportion of original cosponsors on a legislator's bill who are from the opposing party, on average, across all bills the legislator sponsored in a given Congress. Formally, let S_{ijt}^{opp} denote the number of original cosponsors from the opposing party on bill j sponsored by legislator i in Congress t, S_{ijt} denote the total number of original cosponsors on j sponsored by i in t, and J_{it} denote the number of bills sponsored by i in t. Then *Bipartisan Original Cosponsorships Attracted*, A_{it}, is calculated as:

$$A_{it} = \frac{1}{J_{it}} \sum_{j \in J_{it}} \frac{S_{ijt}^{\text{opp}}}{S_{ijt}}. \quad [1]$$

Our measure for *Bipartisan Original Cosponsorships Offered* captures the proportion of bills for which a legislator was an original cosponsor that members of the opposing party introduced. Formally, let C_{it}^{opp} denote the number of original cosponsorships by legislator i on bills introduced by members of the opposing party in Congress t and C_{it} denote the total number of original cosponsorships by i in t. Then *Bipartisanship Original Cosponsorships Offered*, O_{it}, is calculated as:

$$O_{it} = \frac{C_{it}^{\text{opp}}}{C_{it}}. \quad [2]$$

Members who did not sponsor any bills or did not engage in original cosponsorship are assigned a value of zero. Additionally, we measure these outcomes in three successive two-year congressional terms after treatment. In cases where a legislator served in the immediate term but not in the second or third terms (e.g., due to losing a re-election bid), we code the outcome variables based on the legislator who did serve in the second or third term for that district. This approach avoids post-treatment bias from non-random attrition rates between amateurs and incumbents. It also underscores the point noted in the main text and below that we must interpret the results at the district level (5, 6).

Recall from above that we only consider original cosponsors in our main outcome measurement strategy. However, our results are robust to measures of bipartisan collaboration constructed using *all* cosponsorship data, which credits any legislator who ever signed on to a bill as a cosponsor. Data employed in this measurement construction followed identical inclusion criteria, whereby we limited our analysis to bills that received at least one cosponsorship and excluded any withdrawn cosponsorships. Outcome measures employ the same calculation formulas, differing only in their focus on all cosponsorships rather than exclusively original cosponsors. Similarly, our results are robust to outcome measures that reflect raw counts of cross-party cosponsorships, rather than the rate-based measures described above.

Regression discontinuity design. We first analyze the election of amateurs to the U.S. House in a regression discontinuity design (RDD). In brief, our RDD leverages candidates' general election, two-party vote shares—the percentage of votes received by the amateur candidate relative to the total votes cast for the two major parties—as the forcing variable. Treatment assignment occurs as-if randomly at the 50% vote threshold, distinguishing barely victorious amateur candidates from narrowly defeated incumbents (7). Because our treatment is a politician's characteristic (amateur or experienced challenger status), interpreting results at the candidate level risks bias from factors correlated with election competitiveness and subsequent outcomes (5). Thus, our target estimand is the local average treatment effect (LATE) of a congressional district electing an amateur instead of an incumbent (Figure 1, panel a) or an experienced challenger instead of an incumbent (panel b) (6).

Sample inclusion: Our universe of observations includes all contested U.S. House general elections where the top two vote-getters were a Democrat and a Republican. Within these elections, we specifically examine contests between an amateur (or experienced) challenger and an incumbent member of the U.S. House (N = 5,875). This sample of eligible contests is further limited to marginal contests, where an amateur (experienced) candidate either narrowly won or narrowly lost the general election. For the results presented in the main text, we apply the mean squared error (MSE)-optimized bandwidth selector and a triangular kernel. This method selects bandwidths of approximately five percentage points on either side of the threshold for the estimation with amateur candidates (Figure 1, panel a). The effective sample sizes—which account for the bandwidth and weighting scheme—for the amateur results are 276 (cosponsorships attracted) and 254 (cosponsorships offered). The analogous values for the experienced challengers analysis (N = 1,345) reported in Figure 1, panel (b) are as follows: the

MSE-selected bandwidths are ± 0.07 (cosponsorships attracted) and ± 0.08 (cosponsorships offered); the effective sample sizes are 307 (cosponsorships attracted) and 329 (cosponsorships offered).

The RDD samples are broadly representative of the eligible population in terms of demographic, institutional, and ideological characteristics. Cases in both our analyses modestly over-represent two-party competitive races, which is unsurprising given the criteria for sample inclusion. They also slightly over-represent ideologically liberal candidates. These points again highlight the interpretation of the RDD results as the bundled treatment of the political circumstances leading to the election of an amateur (experienced challenger) instead of the incumbent (5, 6).

Estimation: Our RDD estimation involves fitting regressions of the following form:

$$y_{dt} = \mu + \tau \text{Amateur}_{dt} + f(M_{dt}) + \varepsilon_{dt}, \quad [3]$$

in which y_{dt} is an outcome variable representing the cosponsorship behavior of the Congressperson representing district d in congressional term t. Amateur_{dt} is an indicator for an election winner who lacks prior political experience (treatment = 1) or an incumbent (treatment = 0) and $f(M_{dt})$ is a flexible function of the amateur candidate's two-party vote margin. This variable is coded such that zero represents an exact tie and positive (negative) values represent electoral victory (defeat) for the amateur candidate. Our quantity of interest (τ) is the effect of a district electing an amateur who receives marginally more vote share than an exact tie. In our second RDD analysis, we replace the contrast between amateurs and incumbents with that between experienced challengers and incumbents. All other elements of the estimation process remain the same.

Identifying assumptions: To test the RDD identification strategy, we first assess the density of the forcing variable at the threshold. Differences in density above and below the threshold would suggest sorting around the cutoff, signaling potential endogenous influences on outcomes occurring at the same threshold used to determine treatment status (7). A test of the null hypothesis of equal density yields a test statistic of 0.670 (p = 0.503) for the RDD comparing amateurs to incumbents and −0.373 (p = 0.709) for the RDD comparing experienced challengers to incumbents. Thus, we retain the null in both cases.

Next, we assess whether the RDD produces significant differences when employing pretreatment covariates as placebo outcomes. The logic of this falsification test is straightforward: units just above and below the electoral threshold should appear similar on observable covariates. Imbalances in district-level covariates indicate that estimated effects may be due to differences between the types of districts that barely elect an amateur candidate rather than retaining the incumbent (5). Imbalance in candidate characteristics could raise concerns about sorting (6). We assess balance on the following characteristics:

- Vacant seat;
- Total campaign fundraising (logged);
- Southern state;
- District redistricted;
- Northeastern state;
- Midwestern state;
- Lagged House vote;
- Ideology (based on campaign donors);
- Female candidate;
- District total population;
- District median income;
- Average Democratic presidential vote in district;
- Closed primary;
- Percent district white.

The results for both the amateurs and experienced challengers RDDs show balance on all but one covariate in the data. That exception is the lagged vote in the district, which consistently favors incumbents. This difference reflects the pattern that amateurs and experienced challengers often face a particularly challenging campaign environment (2). From an identification standpoint, it again indicates that interpretation of RDD estimates must be made at the district level (5, 6). As noted above, our analyses estimate the effects of the bundle of factors (including historical incumbent performance) that lead an amateur or experienced challenger to break through and (barely) win over an incumbent.

As another test for RDD validity, we estimate treatment effects at placebo thresholds. This procedure assesses continuity in the regression function by examining treated and control units in the absence of treatment. While continuity precisely *at the threshold* is critical for identification, it is inherently untestable. However, evaluating continuity at alternative placebo thresholds provides valuable insight, as discovering discontinuities at these points would cast doubt on the overall validity of the design. We estimate RDD effects at placebo cutoff points—specifically thresholds of 1–10% in increments of 1% in vote margin—-using only treated observations (7). These placebo effects should consistently approximate zero under a valid RDD design, indicating continuity in the outcome variable comparing amateurs who won, respectively, by those levels or more to winners with margins less than those levels. In general, the placebo effects are small in magnitude, statistically indistinguishable from zero, and/or exhibit no discernible systematic patterns, further supporting the credibility of our RDD design.

Finally, to test robustness, we estimate the RDD effects using a variety of specifications for the local regression functions. The results reported in the main text come from a local linear regression specification, a common mean squared error (MSE) optimized bandwidth selector, and triangular kernel. Similar estimates are obtained from local regressions using quadratic,

cubic, or quartic polynomials. Additionally, our substantive conclusions are unchanged if we employ coverage error rate (CER) optimization instead of MSE (8) and if we employ different bandwidths on either side of the threshold. Our results are also substantively similar using a grid search over bandwidths ranging from 3–35% of vote share difference. Finally, the results hold if we condition on the seniority (years served) of a district's member of Congress.

Panel data design. These data exhibit a panel structure, with congressional districts serving as units (i) observed across two-year terms following a general election, which constitute the time periods (t). Our RDD analysis does not emphasize the temporal component; it simply pools elections to improve the statistical power of the estimates. By employing PanelMatch (PM) and the fixed effects counterfactual estimator (FEct), we can leverage this longitudinal structure to examine the dynamic effects of electing amateurs on bipartisan behavior. Moreover, employing panel data estimators allows us to assess the robustness of our conclusions from the RDD analysis to a different set of assumptions.[*] In this design, we define a district-congress observation as treated in all time periods during which the elected representative was an amateur, and untreated otherwise.

Sample inclusion: our universe of observations encompasses all members of the U.S. House of Representatives elected from 1980 to 2022, covering the 97th through the 118th Congresses. The estimator requires that unit and time identifiers correspond uniquely to each observation; however, this condition is violated when multiple incumbents represent a single district within a single congressional term. This situation occurs when an incumbent leaves office and is succeeded through a special election. To address this issue, we excluded such district-congress observations from the main analysis reported in the paper. Including only one incumbent from these duplicated observations (either the original or the successor) yields substantively identical results. The final dataset contains 9,432 rows: 501 unique districts, each appearing in the data for about 19 years, on average.[†]

PanelMatch estimation: we first employ the PM estimator, which identifies effects by adapting the logic of matching methods to panel data (9).[‡] It is a design-based method that permits balance assessment in time-varying covariates and estimation of dynamic treatment effects. It is more robust to model misspecification than the two-way fixed effects regression model (TWFE), though several assumptions are important to note. First, it assumes no spillover effects. In this case, a congressional district's potential outcomes are only affected by its own treatment history up to a specified number of lags. We employ a lag of four periods (congressional terms) to bolster the credibility of this assumption. Additionally, the assumption necessitates that the potential outcomes of one district are not dependent on the treatment status of another. Finally, like TWFE, PM assumes parallel trends. We discuss the empirical assessment of this assumption below.

The method begins by identifying a set of control districts for each treated district that carry identical treatment histories as the treated district for a user-defined number of time periods. Specifically, the method constructs a matched set of control units ($\mathcal{M}$) for each treated case that shares treatment history (x) based on a lag length (L) selected by the user (9):

$$\mathcal{M}_{it} = i' : i' \neq i, x_{i't} = 0, x_{i't'} = x_{it'} \\ \forall t' = t-1, \ldots, t-L. \quad [4]$$

Specifying a longer lag strengthens the credibility of the design, but reduces efficiency because finding matches becomes more difficult. We are able to use four periods of treatment history for this step.

This process matches treatment history, but does not account for covariates. The method offers several options for further refining the matched sets through an additional matching step or weighting to improve covariate balance. In this case, we select weighting with the covariate balancing propensity score (CBPS) methodology (11), although results do not depend on this choice. We include the following covariates for this estimation:

- Candidate ideology estimated from campaign donations;
- Seniority, measured as years served in Congress;
- Candidate gender;
- Candidate race;
- An indicator for a newly-created district due to redistricting;
- An indicator for candidates with no prior *legislative* experience;
- Indicators for congressional leadership (party leaders and committee chairs);
- Democratic average presidential vote in the district over the last two election cycles;
- District population, proportion white, and median income;
- The size (in seats) of the legislative majority for a given Congress; negative values indicate minority party status.

Diagnostics indicate that nearly all covariates are balanced with CBPS weighting, defined as standardized mean differences at each of the four pretreatment lags being smaller than 0.20 in absolute value. The exceptions are candidate gender and race.

Finally, PM implements a difference-in-differences estimator on the matched sets (which now account for treatment history and covariates) for a user-defined number of time points in the future (we choose three periods). This estimator controls for time-invariant characteristics of districts as well as potential shocks that affect all districts at a given point in time. We select the average treatment effect on the treated (ATT) as the target estimand. The standard errors are computed using a block bootstrap, in which districts are resampled over 1,000 iterations.

[*] These methods rely on covariate adjustment rather than an as-if random treatment assignment mechanism like RDD. Thus, our causal leverage is reduced in the panel data analyses.
[†] About half of the districts (270) appear in all 22 time periods, but others appear in only some years due to redistricting.
[‡] This summary of PM is based on a the discussion of the estimator contained in the SI of (10).

FEct estimation: we also use FEct estimated by matrix completion (MC) in our panel data analyses (9, 12). This method belongs to a class of "imputation estimators" for panel data that improves on the logic of the two-way fixed effects estimator—a regression model with indicators for units and time periods. FEct fits a parametric model with unit (α_d) and time (ξ_c) fixed effects, and time-varying covariates (x) to the untreated observations in the sample to estimate the potential outcomes under control.[§] The covariates included in the specifications are identical to those used in PM (see above).

The MC version of the estimator further conditions on $\mathbf{L}$, a low-rank matrix that represents time-varying unobserved factors extracted from the data but not captured in x_{dc} (9, 12):

$$y_{dc}(0) = x'_{dc}\beta + \alpha_d + \xi_c + \mathbf{L} + \varepsilon_{dc}. \qquad [5]$$

FEct then imputes $y_{dc}(0)$ for the treated observations using the model in Equation 5 and computes period-wise treatment effects with the differences between the actual and imputed outcomes (9, 13). Under a functional form assumption for the imputation model and exogeneity of treatment conditional on the fixed effects, covariates, and $\mathbf{L}$, this process identifies the average treatment effect on the treated (ATT) in each time period (13). We employ this estimator with each outcome variable.

A key consideration when employing FEct is the plausibility of its identifying assumptions. To assess these assumptions empirically, we perform equivalence tests of pretreatment trends and placebo tests examining anticipatory (pretreatment) effects (9). These diagnostics, which we discuss here, collectively support the validity of using FEct in our panel data analysis.

The pretreatment estimates represent average differences between observed outcomes and predictions generated by the FEct model using untreated observations within each time period. Under the assumption of no pretreatment trend—a critical component of the estimator's strict exogeneity assumption—these estimates should be zero in expectation (9, 13). To investigate this assumption, we compute the p-value associated with an equivalence test for a pretreatment trend. Specifically, we conduct a two one-sided test (TOST) by assessing whether the pretreatment estimates' 90% confidence intervals fall within an equivalence range, defined as 0.15σ, where σ is the outcome standard deviation net of the unit and time effects.[¶] The obtained p-value of 0.000 rejects the null hypothesis that the pretreatment estimates differ significantly from zero.

We further evaluate the FEct identification assumptions using a placebo test to detect potential anticipatory effects of treatment. Specifically, we exclude the two time periods immediately preceding treatment from model estimation and instead estimate placebo effects for these held-out periods. Under a valid research design, these placebo estimates should be zero in expectation. The associated p-value tests the null hypothesis that the placebo estimates are jointly nonzero, with smaller values indicating greater support for the design's validity. In our data, the analyses of both outcome variables yield placebo p-values of 0.000, providing additional evidence in favor of our panel design.

Alternative Outcome: Total Cosponsorships

Another outcome variable that can lend insight into our research question is the *total number* of cosponsorships attracted and offered—the denominator of the proportion used in our main outcomes. The intuition is that amateur politicians may participate less in the legislative process overall, rather than specifically avoiding bipartisan collaboration. In other words, amateurs might fail to comprehend the legislative process more generally rather than the value of bipartisanship specifically. To test this possibility, we replicate the main analyses (RDD, PM, and FEct) using the total volume of original cosponsorships attracted/offered as the outcome.

The results provide only limited support for the logic outlined above. In nearly all cases, the effects on cosponsorship volume are close to zero and not statistically distinguishable from zero. The one exception is the FEct estimates on total cosponsorships offered, which indicate a negative and statistically significant effect of approximately five bills in the first session after an amateur is elected. This result is consistent with the idea that amateurs may lack a general understanding of the legislative process. However, this result does not replicate in the RDD or PanelMatch results, nor with the cosponsorships attracted outcome. Taken together, the evidence suggests that a lack of appreciation for the value of bipartisanship—rather than a broader unfamiliarity with legislative procedure—is the distinguishing feature of amateur lawmakers.

Meta-Analysis of Bipartisanship Effect Magnitudes

What is the economic significance of electing amateur politicians for cross-party cooperation? Do the negative effects we report extrapolate to a meaningful or trivial reduction in collaboration across the aisle? To contextualize our findings, we benchmark our estimates against prominent studies that have evaluated individual, district, and institutional-level determinants of bipartisan legislative behavior in the U.S. Congress. To facilitate meaningful comparisons, we restrict our attention to research employing outcome variables closely aligned with ours: the proportion of cross-party cosponsorships attracted or offered. We specifically compare these effects to the following results from past work, with effect sizes provided in brackets that are on the same scale as our main analysis effects presented in the paper.

- **2020 election denier**: Republican MCs who voted against the certification of electoral votes for the 2020 presidential election on January 6, 2021, experienced a *decrease in Democratic cosponsorships attracted to their legislation* compared to those who did not vote against certification (15). **[−0.073 decrease in other-party cosponsorships attracted]**

[§] We set the estimator to require a minimum of three time periods of data prior to the election of an amateur for inclusion in the estimation and a maximum of six such periods for use in evaluating model fit. Results are not sensitive to these choices.

[¶] This standard is *stricter* than conventional practice (9, 14).

- **Bipartisan congressional travel**: MCs who participate in official congressional delegation (CODEL) trips with colleagues from the opposing party *attract more opposing-party cosponsorships* compared to those who do not engage in bipartisan travel (16). **[+0.002 increase in other-party cosponsorships attracted]**
- **Effective lawmaker**: MCs with higher legislative effectiveness ratings *attract more bipartisan cosponsorships* compared to less-effective legislators, although there is *no difference in bipartisan cosponsorships offered* (17).[‖] **[+0.013 increase in other-party cosponsorships attracted]**
- **Extreme district**: MCs representing districts with a greater proportion of same-party identifying constituents *offer bipartisan cosponsorships less frequently* compared to those representing districts with an even distribution (18). **[−0.018 decrease in other-party cosponsorships offered]**
- **Local roots representative**: MCs who represent the district in which they were born *attract more bipartisan cosponsorships* compared to those who do not represent the district in which they were born (19). **[+0.030 increase in other-party cosponsorships attracted]**
- **Military veteran**: MCs who served in the U.S. military *offer more bipartisan cosponsorships* compared to a lawmaker who did not serve in the military (20). **[−0.011 decrease in other-party cosponsorships offered]**

Comparing these estimates shows that the effects of electing amateurs on bipartisan collaboration are substantively meaningful. We benchmark our effect sizes (RDD, PM, FEct) against those reported in the studies referenced above, expressing them as ratios that indicate how much larger or smaller our effects are relative to prior findings. For instance, the RDD estimate for other-party original cosponsorships attracted is more than two and a half times larger than the effect reported for being a 2020 election denier. A complete set of conversions is presented in the bulleted list below:

- **2020 election denier** (attracted): 2.59 (RDD); 1.17 (PM); 0.38 (FEct)
- **Bipartisan congressional travel** (attracted): 94.50 (RDD); 42.79 (PM); 14.03 (FEct)
- **Effective lawmaker** (attracted): 14.54 (RDD); 6.58 (PM); 2.16 (FEct)
- **Extreme district** (offered): 12.28 (RDD); 2.76 (PM); 2.17 (FEct)
- **Local roots representative** (attracted): 6.30 (RDD); 2.85 (PM); 0.94 (FEct)
- **Military veteran** (offered): 20.09 (RDD); 4.51 (PM); 3.56 (FEct)

References

1. GC Jacobson, Strategic politicians and the dynamics of US House elections, 1946–86. *Am. Polit. Sci. Rev.* **83**, 773–793 (1989).
2. R Porter, SA Treul, Evaluating (in)experience in congressional elections. *Am. J. Polit. Sci.* **69**, 284–298 (2025).
3. A Bonica, Database on ideology, money in politics, and elections: Public version 3.1 [computer file]. (2023) Stanford, CA: Stanford University Libraries. https://data.stanford.edu/dime.
4. JT Ornstein, Probabilistic record linkage using pretrained text embeddings (2025) Presented at the Society of Political Methodology Annual Conference, University of California–Riverside.
5. J Marshall, Can close election regression discontinuity designs identify effects of winning politician characteristics? *Am. J. Polit. Sci.* **68**, 494–510 (2024).
6. A Bertoli, C Hazlett, Seeing like a district: Understanding what close-election designs for leader characteristics can and cannot tell us. *Polit. Analysis* (2025, https://doi.org/10.1017/pan.2025.5).
7. MD Cattaneo, N Idrobo, R Titiunik, *A Practical Introduction to Regression Discontinuity Designs: Foundations*, Elements in Quantitative and Computational Methods for the Social Sciences. (Cambridge University Press, New York), (2020).
8. L De Magalhães, et al., When can we trust regression discontinuity design estimates from close elections? Evidence from experimental benchmarks. *Polit. Analysis* **33**, 258–265 (2025).
9. L Liu, Y Wang, Y Xu, A practical guide to counterfactual estimators for causal inference with time-series cross-sectional data. *Am. J. Polit. Sci.* **68**, 160–176 (2024).
10. JJ Harden, A Campos, Who benefits from voter identification laws? *Proc. Natl. Acad. Sci.* **120**, 1–3 (2023).
11. K Imai, M Ratkovic, Covariate balancing propensity score. *J. Royal Stat. Soc. Ser. B (Stat. Meth.)* **76**, 243–263 (2014).
12. S Athey, M Bayati, N Doudchenko, G Imbens, K Khosravi, Matrix completion methods for causal panel data models. *J. Am. Stat. Assoc.* **116**, 1716–1730 (2021).
13. A Chiu, X Lan, Z Liu, Y Xu, Causal panel analysis under parallel trends: Lessons from a large reanalysis study. *Am. Polit. Sci. Rev.* (2025, https://doi.org/10.1017/S0003055425000243).
14. E Hartman, FD Hidalgo, An equivalence approach to balance and placebo tests. *Am. J. Polit. Sci.* **62**, 1000–1013 (2018).
15. JM Curry, JM Roberts, Interpersonal relationships, bipartisanship, and January 6th. *Am. Polit. Sci. Rev.* **119**, 1542–1548 (2025).

[‖] These results come from a replication and extension of the original study, which focuses on bipartisanship as a predictor and legislative effectiveness as the outcome variable.

16. JM Curry, JM Roberts, Interpersonal relationships and legislative collaboration in congress. *Legislative Stud. Q.* **48**, 333–369 (2023).
17. L Harbridge-Yong, C Volden, AE Wiseman, The bipartisan path to effective lawmaking. *J. Polit.* **85**, 1048–1063 (2023).
18. L Harbridge, N Malhotra, Electoral incentives and partisan conflict in congress: Evidence from survey experiments. *Am. J. Polit. Sci.* **55**, 494–510 (2011).
19. J Crosson, J Kaslovsky, Do local roots impact washington behaviors? district connections and representation in the u.s. congress (2024) Forthcoming, *American Political Science Review.* https://doi.org/10.1017/S000305542400056X.
20. JG Amoroso, Deployed to the hill: Military experience and legislative behavior in congress. *Polit. Res. Q.* **78**, 358–375 (2024).
